# Inorganic Fe-O and Fe-S oxidoreductases: paradigms for prebiotic chemistry and the evolution of enzymatic activity in biology

**DOI:** 10.3389/fchem.2024.1349020

**Published:** 2024-02-08

**Authors:** Xiao-Lan Huang, Jeffrey R. Harmer, Gerhard Schenk, Gordon Southam

**Affiliations:** ^1^ NYS Center for Clean Water Technology, School of Marine and Atmospheric Sciences, Stony Brook, NY, United States; ^2^ Australian Institute of Bioengineering and Nanotechnology, The University of Queensland, Brisbane, QLD, Australia; ^3^ School of Chemistry and Molecular Biosciences, The University of Queensland, Brisbane, QLD, Australia; ^4^ Sustainable Minerals Institute, The University of Queensland, Brisbane, QLD, Australia; ^5^ School of the Environment, The University of Queensland, Brisbane, QLD, Australia

**Keywords:** oxidoreductases, biocatalysts, inorganic nanocatalysts, metal ion architecture, evolution, sustainability, biotechnology

## Abstract

Oxidoreductases play crucial roles in electron transfer during biological redox reactions. These reactions are not exclusive to protein-based biocatalysts; nano-size (<100 nm), fine-grained inorganic colloids, such as iron oxides and sulfides, also participate. These nanocolloids exhibit intrinsic redox activity and possess direct electron transfer capacities comparable to their biological counterparts. The unique metal ion architecture of these nanocolloids, including electron configurations, coordination environment, electron conductivity, and the ability to promote spontaneous electron hopping, contributes to their transfer capabilities. Nano-size inorganic colloids are believed to be among the earliest ‘oxidoreductases’ to have ‘evolved’ on early Earth, playing critical roles in biological systems. Representing a distinct type of biocatalysts alongside metalloproteins, these nanoparticles offer an early alternative to protein-based oxidoreductase activity. While the roles of inorganic nano-sized catalysts in current Earth ecosystems are intuitively significant, they remain poorly understood and underestimated. Their contribution to chemical reactions and biogeochemical cycles likely helped shape and maintain the balance of our planet’s ecosystems. However, their potential applications in biomedical, agricultural, and environmental protection sectors have not been fully explored or exploited. This review examines the structure, properties, and mechanisms of such catalysts from a material’s evolutionary standpoint, aiming to raise awareness of their potential to provide innovative solutions to some of Earth’s sustainability challenges.

## 1 Introduction-inorganic abiotic nanocolloids as efficient catalysts of biologically relevant reactions

Oxidoreductases are a superfamily of enzymes (*i.e.,* biocatalysts) found throughout the tree of life (Williams, 1981; Falkowski et al., 2008; Kim et al., 2013). These enzymes are molecular machines responsible for virtually all biologically induced electron transfer (ET) reactions. Examples include peroxidases (PODs), catalases (CATs), superoxide dismutases (SODs) and oxidases (OXDs). Various metabolic pathways, such as glycolysis, the Krebs cycle, photosynthesis in chloroplasts, drug metabolism and detoxification reactions in the liver require oxidoreductases. Reactive oxygen species (ROS) and hydrogen peroxide (H_2_O_2_) are frequently observed metabolites in reactions catalyzed by oxidoreductases (Apel and Hirt, 2004; Bayr, 2005; Valko et al., 2007; Sharma et al., 2012). PODs use H_2_O_2_ or organic hydroperoxides (R-OOH) as electron donors and H_2_O_2_ as electron acceptor during redox reactions (Rodríguez-López et al., 2001; Veitch, 2004; Leblanc et al., 2015; de Oliveira et al., 2021). OXDs catalyze the oxidation of various substrates (electron donors) by using molecular oxygen (O_2_) as an electron acceptor. In these reactions, hydrogen atoms are used to form water or H_2_O_2_ by enzymes such as sulfite oxidase (SOE), glucose oxidase (GOX), or alcohol oxidase (AOX) (Messner and Imlay, 2002; Leskovac et al., 2005; Jancura et al., 2014; Kappler and Enemark, 2015). CATs accelerate the decomposition of H_2_O_2_ into water and O_2_ (Deisseroth and Dounce, 1970; Alfonso-Prieto et al., 2009), while SODs disproportionately divide superoxide radicals (O_2_^*−^) into H_2_O_2_ and O_2_ (Fridavich, 1995; Sheng et al., 2014).

The primary function of biological oxidoreductases is ET, though some oxidoreductases can transfer electrons directly or through mediators such as cytochrome *c* (Cyt *c*), to solid surfaces, including electrodes, enzymes, microorganisms and nanomaterials (Kracke et al., 2015; Milton and Minteer, 2017; Chen H. et al., 2020; Ratautas and Dagys, 2020). This process, known as direct ET (DET) (Kracke et al., 2015; Milton and Minteer, 2017; Chen H. et al., 2020; Ratautas and Dagys, 2020; Suprun, 2021) was first observed in 1977 (Eddowes and Hill, 1977; Peter and Theodore, 1977) for Cyt *c* on gold and tin-doped indium oxide electrodes, exhibiting virtually reversible electrochemistry as revealed by cyclic voltammetry. Horseradish peroxidase **(**HRP) (Yaropolov et al., 1979) and laccase (Lc) (Tarasevich et al., 1979) have been shown to adsorb on carbon electrodes and exhibit DET capacity. Currently, more than 100 enzymes are known to be capable of working under DET conditions, with the majority being oxidoreductases (Gorton et al., 1999; Ferapontova et al., 2003; Shleev et al., 2005; Liu et al., 2006; Léger and Bertrand, 2008; Liu et al., 2014; Bollella et al., 2018).

Oxidoreductase activity is not limited to protein-based catalysts; some inorganic colloids with oxidoreductase-like activity are able to catalyze biochemical reactions *in vitro* and *in vivo* (Wei and Wang, 2013; Wu J. et al., 2019; Huang et al., 2019; Liang and Yan, 2019; Singh, 2019; Zhang X. et al., 2021; Yang et al., 2021; Hong et al., 2022). It should be noted that inorganic colloids can perform other catalytic functions, including the hydrolysis of phosphate ester bonds (Huang and Zhang, 2007; Huang and Zhang, 2012; Huang, 2018; Huang, 2019)*.* Some of the best studied inorganic systems are iron oxides such as inorganic peroxidase (*e.g.*, magnetite (Mag, Fe_3_O_4_) colloids (1-1,000 nm)) that can include a highly reactive nanoparticle (NP) sub-fraction (<100 nm) (Gao et al., 2007; Chaudhari et al., 2012; Chen et al., 2012; Gao et al., 2017; Gao and Yan, 2019; Gao, 2022). Synthetic Mag NPs were the first inorganic nanomaterials reported to possess intrinsic POD-like properties (Gao et al., 2007) catalyzing the oxidation of organic substrates such as 3,3,5,5-tetramethylbenzidine (TMB), diazoaminobenzene (DAB) and o-phenylenediamine (OPD). Displaying Michaelis-Menten-type behavior, their reaction velocity is inversely related to the particle size (*i.e.*, the larger the surface area of the NPs/colloids the greater their activity) (Gao et al., 2007). In terms of their catalytic efficiency (*k*_cat_/*K*_m_) some of these abiotic catalysts (H_2_O_2_: 560 mM^-1^ s^-1^; TMB: 3.1×10^5^ mM^-1^ s^-1^) are comparable to their biological counterparts (H_2_O_2_: 940 mM^-1^ s^-1^; TMB: 9.2×10^3^ mM^-1^ s^-1^) (Gao et al., 2007). Numerous iron oxide colloids have been shown to exhibit similar intrinsic POD activity, including maghemite (Mah, γ-Fe_2_O_3_) (Chen et al., 2012), hematite (Hem, α-Fe_2_O_3_) (Chaudhari et al., 2012), two-dimensional lepidocrocite nanomaterials formed from graphene-templates (Peng et al., 2011), and Prussian blue-modified iron oxide magnetic compounds (Wang and Huang, 2011). These inorganic catalysts also display substrate selectivity, temperature responsiveness and pH dependence similar to natural enzymes (Gao et al., 2007; André et al., 2011; Huang and Zhang, 2012; Wei and Wang, 2013; Wu et al., 2019; Huang, 2018, 2019, 2022a). This observation has the potential to revolutionize various industries and applications, offering more efficient and customized catalytic processes. The implications for fields such as medicine (Gao and Yan, 2019; Lopez-Cantu et al., 2022; Wei et al., 2023), environmental science (Meng et al., 2020; Wong et al., 2021), and agricultural production (Liu et al., 2021; Cui et al., 2022) are truly exciting.

Inorganic nanocatalysts, possessing enzyme-like activity are not limited to iron oxides and sulfides, *i.e.*, many other metal NPs exhibit properties or functions similar to enzymes. For example, molybdenum disulfide (MoS_2_) NPs possess both semiconductor properties (Radisavljevic et al., 2011) and electron hopping behavior (Qiu et al., 2013), allowing them to naturally act as POD, CAT, and SOD (Chen et al., 2018; Yu et al., 2021). Similarly, mixed-valence vanadium pentoxide V_2_O_5_ NPs exhibit semiconducting characteristics (Sanchez et al., 1983a) due to electron hopping dynamics within V^4+^ and V^5+^ ions (Sanchez et al., 1983b), and also exhibit intrinsic POD, GOX and glutathione peroxidase (GPx) activity (André et al., 2011; Natalio et al., 2012; Ghosh et al., 2018; Ding Y. et al., 2020; Chen, 2022). In MnO_2_ NPs, direct electron hops within Mn - Mn chains (Devaraj and Munichandraiah, 2008; Farooq et al., 2019) result in POD, CAT, OXD, and SOD activities (Huang Y. et al., 2016; Tang et al., 2022), whereas Co_3_O_4_ NPs exhibit semiconducting attributes marked by Co^3+^-Co^2+^ hopping (Cheng et al., 1998; Pham et al., 2016; Ibrahim et al., 2018), enabling intrinsic POD and CAT activities (Mu et al., 2012; Mu et al., 2014; Li et al., 2018; Wang et al., 2018). Other NPs like α-FeSe, and Cu_2_O/CuO, known for their superconductivity (Ito et al., 1991; Hsu et al., 2008; Sidorov et al., 2011; Lai et al., 2015), also demonstrate intrinsic POD activity (Dutta et al., 2012a; Dutta et al., 2012b; Dutta et al., 2013; Liu T. et al., 2020; Jiang et al., 2021; Zhu et al., 2021). NPs with lower bandgaps and electron hopping, such as titanium dioxide (TiO_2_) (Setvin et al., 2014; Xu Z. F. et al., 2022), manganese selenide (MnSe) (Liu et al., 2023), and molybdenum selenide (MoSe_2_) (Suri and Patel, 2017), also display intrinsic POD activity (Zhang et al., 2013; Qiao et al., 2014; Wu et al., 2017). An interesting case are nanocrystalline cerium oxide NPs (ceria, CeO_2_), which, due to their high electron conductivity and hopping attributes (Tuller and Nowick, 1977; Kim and Maier, 2002), can directly convert Ce^4+^ to Ce^3+^ due to oxygen vacancies (Esch et al., 2005). This enables ceria NPs to function like oxidoreductases (POD, CAT, OXD, SOD) (Yang et al., 2016a; Montini et al., 2016; Chen et al., 2022; Ma et al., 2022; Xiao et al., 2022), but also like nucleases, phosphatases and photolyases (Zhu et al., 2008; Dhall et al., 2017; Tan et al., 2020a; Tan et al., 2020b)

This review aims to deepen our understanding of the processes that led to the emergence of life on Earth. By bridging the disciplines of inorganic chemistry and biology, we highlight the potential role of inorganic nano-materials in catalyzing complex enzyme-like pre-biotic chemical processes. We propose that these inorganic NPs could have served as the initial biocatalysts for the emergence of the first life forms and subsequent evolutionary processes. This hypothesis challenges established concepts in modern biology, chemistry, and science as a whole. In Section 2 and Section 3, we highlight how the metallic architecture of NPs and their electron hopping characteristics contribute to enzyme-like activity. The physical properties related to ET are foundational to the activity of NPs and may have been crucial in the emergence of life. In Section 4 we will discuss the relevance of such catalytically active NPs in a biological context.

## 2 Architectural changes of iron nanocolloids and their impact on catalytic activity

**FIGURE 1:**
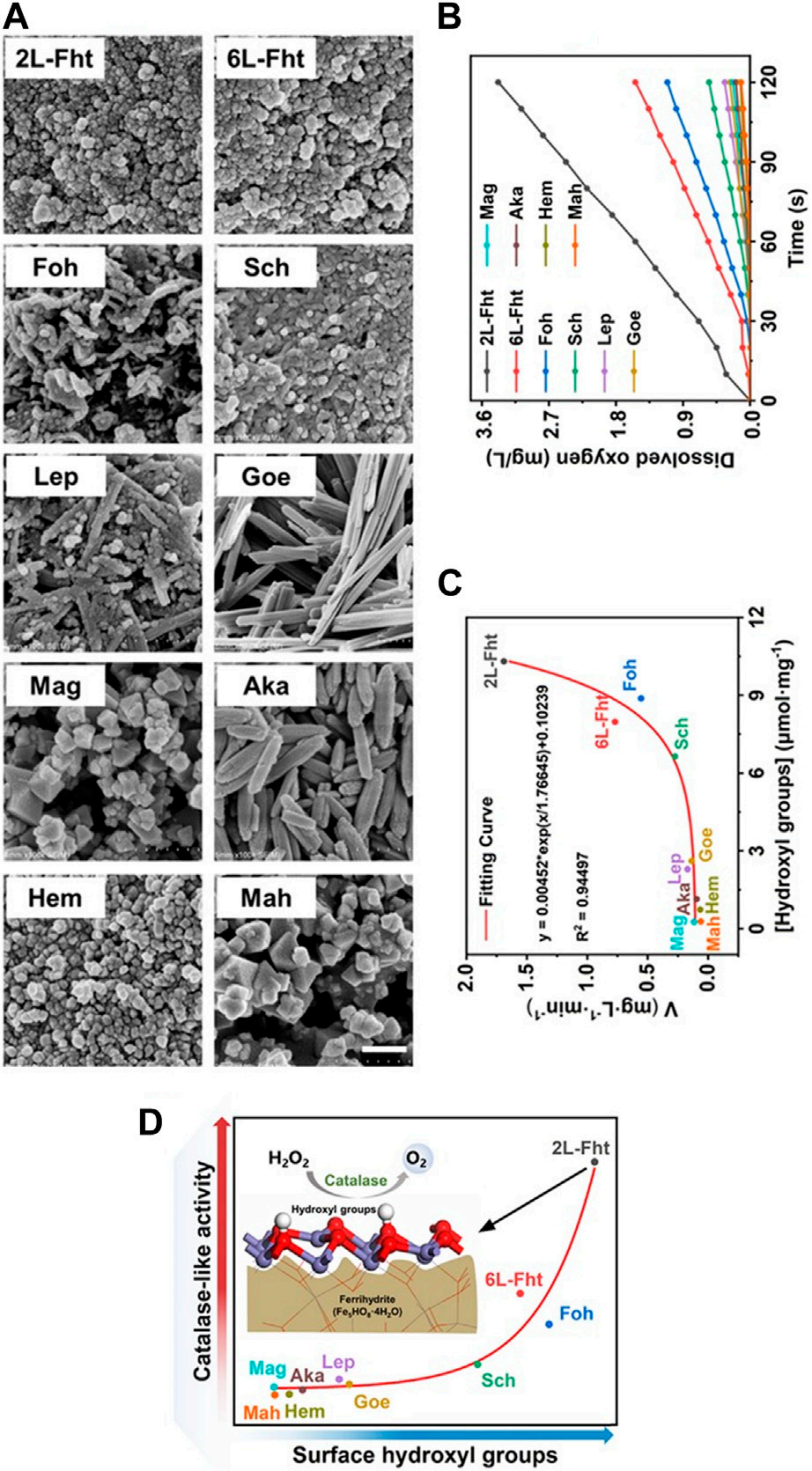
Comparison of the CAT-like activity of ten iron oxide colloids. **(A)** SEM images of ten different iron oxide colloids synthesized using the methods of Cornell and Schwertmann (Cornell and Schwertmann, 2003), including 2L-Fht, 6L-Fht, Goe, Aka, Lep, Foh, Hem, Mah, Mag and Sch. Scale bar, 200 nm. **(B)** Time course for O_2_ formation in a 100 mM H_2_O_2_ solution at 37°C containing various iron oxide colloids (10 μg/mL). **(C)** The number of hydroxyl groups on the surface of iron oxide colloids, measured by acid-base titrations (Tamura et al., 1999) correlates positively with the corresponding catalase-like activities. **(D)** Correlation between CAT activity and surface hydroxyl groups for ten iron oxide colloids. Reprinted from Zhang R. et al., 2021.

Iron oxide systems with CAT-like activity are excellent model systems to illustrate the connection between their architecture and activity. The CAT-like activity of ten synthetic oxide colloids, *i.e.,* 2-line ferrihydrite (2L-Fht, Fe_5_HO_8_·4H_2_O), 6-line ferrihydrite (6L-Fht, Fe_5_HO_8_·4H_2_O), goethite (Goe, α-FeOOH), akageneite (Aka, β-FeOOH), lepidocrocite (Lep, γ-FeOOH), feroxyhyte (Foh, δ′-FeOOH), Hem (α-Fe_2_O_3_), Mah (γ-Fe_2_O_3_), Mag (Fe_3_O_4_) and schwertmannite (Sch, Fe_8_O_8_(OH)_6_SO_4_) (Figure 1A) were compared by monitoring the molecular oxygen they produce in an aqueous H_2_O_2_ solution over time (Figure 1B) (Zhang R. et al., 2021). The activity was found to depend on the number of hydroxyl groups on the surface of the iron oxide colloids (Figure 1C) (Zhang R. et al., 2021).

Iron oxide colloids that have hydroxyl groups in their core structures (2L-Fht, 6L-Fht, Foh) all have significant CAT-like activity, while those that do not (*e.g.,* Hem, Mag and Mah) have no or little activity (Figure 1D) (Zhang R. et al., 2021). Some iron oxide colloids exhibit catalytic promiscuity by having not only CAT-like, but also OXD- and SOD-like activities, all associated with hydrogen peroxide and free oxygen radicals (Chen et al., 2012; Guo and Guo, 2019; Qin et al., 2019; Gu Y. et al., 2020; Chong et al., 2021; Zhao et al., 2021; Xu D. et al., 2022; Gao, 2022). Ferrihydrites, in particular, have high intrinsic CAT-like activity but low intrinsic POD-like activity due to the higher abundance of hydroxyl groups in their crystalline structure compared to other iron oxide colloids (Zhang R. et al., 2021).

Iron oxide nanocolloids^1^ with intrinsic oxidoreductase activity are not limited to synthetic compounds. Inherent oxidoreductase activity has also been observed in Mag from magnetotactic bacteria (MTB) after removing the magnetosome protein membrane (Hu et al., 2010; Li et al., 2015). Biogenic iron oxide colloids from Burkholderia sp. YN01v (Fe_3_O_4_) (Pan et al., 2015; Pan et al., 2019), *Comamonas testosterone* (Fe_1.44_O_0.32_(OH)_3.86_) (Ahmed et al., 2019) and *Acinetobacter* strains (Fe_0.96_O_0.88_(OH)_1.12_) (Abagana et al., 2022) also exhibit intrinsic POD (Pan et al., 2015; Ahmed et al., 2019; Abagana et al., 2022), SOD (Pan et al., 2019) and CAT-like (Pan et al., 2019) activities. The mineral core of ferritin also exhibits POD activity that follows Michaelis-Menten-type kinetics for the oxidation of TMB, OPD and N, N-diethyl-1,4-phenylenediamine (DPD) (Arapova et al., 1999; Tang et al., 2011), as well as SOD activity (Zhang J. et al., 2021). A recent study also shows that the iron cores of various ferritins (Archaea: *Pyrococcus furiosus, Pyrococcus yayanosii,* and *Sulfolobus solfataricus*; Bacteria: *Escherichia coli*; Eukaryotes: *Homo sapiens*) exhibit oxidoreductase activity (POD, CAT, OXD, and SOD) after protein removal (Ma et al., 2024). This activity is attributed to their metal structure rather than the organic compounds in ferritins, particularly the amino acid sequences (Ma et al., 2024).

Iron sulfide nanocolloids have also been reported to have intrinsic oxidoreductase activity, similar to biological oxidoreductases that contain iron-sulfur (Fe-S) clusters, such as alkyl hydroperoxide reductase (Poole, 1996; Hall et al., 2011), disulfide bond oxidoreductase D, rubredoxin or Rieske dioxygenases (Katzen and Beckwith, 2000; Krupp et al., 2001). Furthermore, Fe-S suspensions were shown to catalyze the oxidation of POD substrates such as TMB in the presence of peroxide (Dai et al., 2009; Dutta et al., 2012b). The apparent *K*_m_ values of Fe_7_S_8_ nanowires for H_2_O_2_ and TMB are 0.895 mM and 0.548 mM, respectively, and the corresponding *K*_m_ values of HRP are 0.834 and 3.386 mM, demonstrating again that simple inorganic structures can have substrate affinities that are at least as strong as those of biological representatives (Yao et al., 2013). Greigite nanocolloids (Fe^2+^Fe^3+^_2_S_4_, structural equivalents of Mag) also possess POD-like activity with a high affinity for H_2_O_2_ (Ding et al., 2016; Liu W. et al., 2020). In addition, a nano-colloidal pyrite compound (“pyrite nanozyme”) has recently been shown to have a 3300-fold higher affinity for H_2_O_2_ than Mag, with a more than 4000-fold higher catalytic activity (Meng et al., 2021). It has also been shown that iron polysulfide particles possess POD, CAT and intrinsic glutathione oxidase (GSH-OXD)-like activity (Xu et al., 2018; Cao et al., 2023). These iron sulfide colloids can decompose H_2_O_2_ into free radicals and O_2_, promoting the release of polysulfides. Similar to CAT-, OXD- or SOD-catalyzed reactions various reactive oxygen species (such as hydroxyl (^•^OH), hydrogen peroxide (H_2_O_2_), superoxide (^•^O_2_*) and singlet oxygen (^1^O_2_) are formed in reactions catalyzed by these colloids (Kantar et al., 2019; Nie et al., 2019; Ding W. et al., 2020; Agnihotri et al., 2020; Wang et al., 2020; Huang et al., 2021; Ren et al., 2021; Song et al., 2022). Since most of these ROS trigger cytotoxic effects, metal sulfide nanocolloids may provide a novel therapeutic function (Yuan et al., 2020; Shan et al., 2022).

**FIGURE 2:**
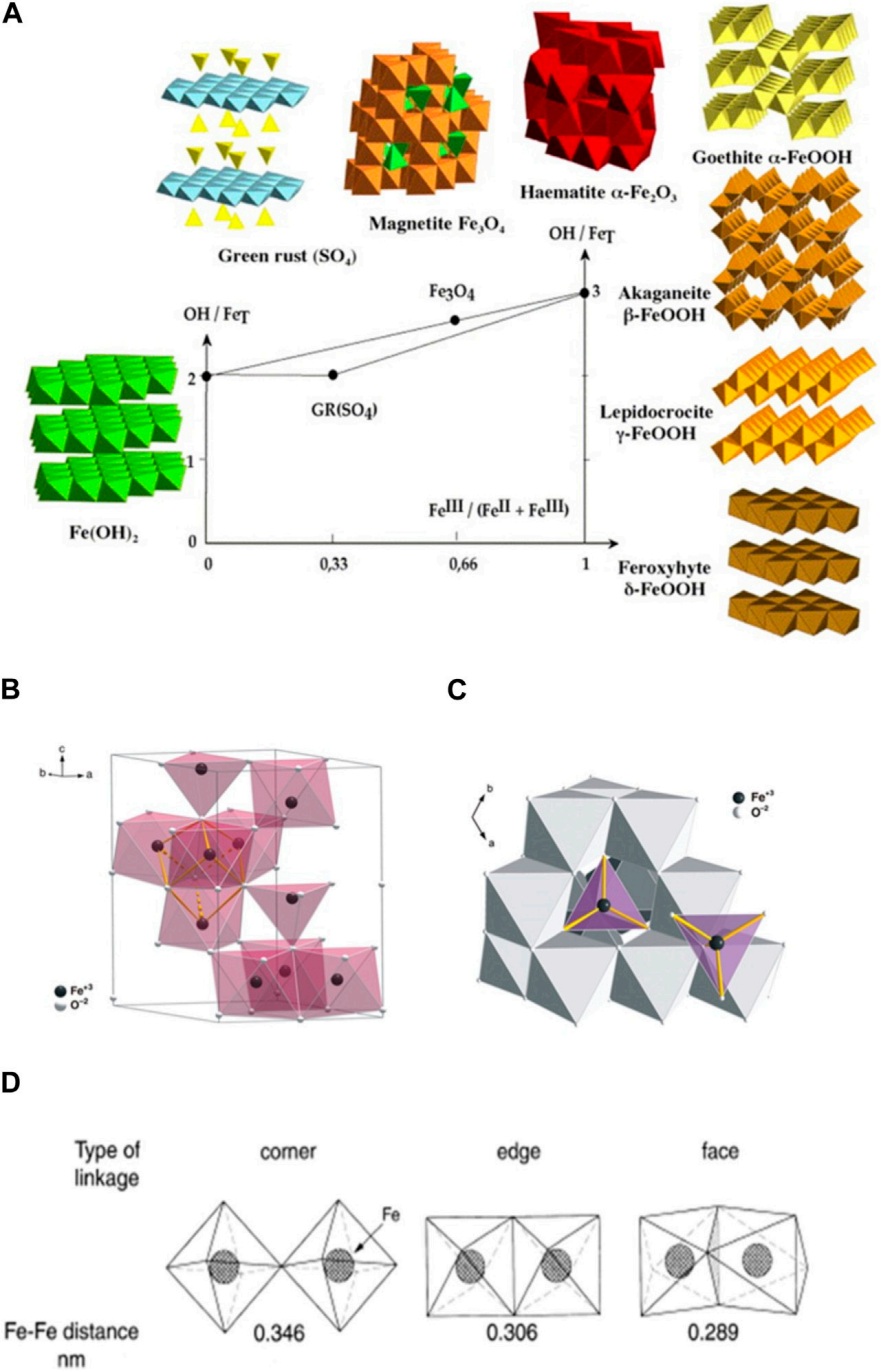
Metal architecture of iron oxide colloids. **(A)** Main structure types of iron oxides (Jolivet et al., 2006) (reprinted with permission from Dr. Jolivet). **(B)** The Back-Figges δ-Keggin Fe_13_ cluster. Polyhedral representation of the ideal ferrihydrite structure viewed along the c axis. The central FeO_4_ tetrahedra are surrounded by 12 FeO_6_ octahedra. **(C)** The basic structural motif consists of a central FeO_4_ tetrahedron surrounded by 12 FeO_6_ octahedra The bonded atoms (yellow) define a cubane-like moiety that connects the basic structural motifs of the model (reprinted from Michel et al., 2007, Copyright ^©^ 2007, AAAS). **(D)** The Fe-Fe distance and linkage of octahedra in Fe^3+^ oxides (reprinted from Cornell and Schwertmann (2003). Copyright @ 2003 John Wiley and Sons).

In addition to size, shape and surface area, recent data indicate that the metal architecture of nanocolloids, including iron oxides, plays a crucial role in enzyme-like activities associated with ET functions (Liu et al., 2011; Puvvada et al., 2012; Cheng et al., 2014; Mu et al., 2014; Peng et al., 2015; Ghosh et al., 2018; Xu Z. et al., 2021; Zhang R. et al., 2021; Jiang et al., 2021; Chen et al., 2022; Singh et al., 2022; Zhang et al., 2022). In general, the metal architecture of iron oxides is determined by their ferric-ferrous composition (*e.g*., Fe^3+^/Fe total) and the hydroxylation ratio (OH/Fe total), as illustrated in Figure 2A (Cornell and Schwertmann, 2003; Jolivet et al., 2006; Jolivet, 2019). As an example, Figure 2B shows the basic structural unit of 2L-Fht/6L-Fht and other iron oxide colloids in a Back-Figges δ-Keggin cluster (Fe_13_), which contains 13 iron and 40 oxygen atoms (Michel et al., 2007; Michel et al., 2010). The central, tetrahedrally coordinated Fe is connected to 12 peripheral, octahedrally coordinated Fe atoms arranged in edge-sharing groups of three by oxo bridges. In this arrangement, iron oxide nanocolloids between 2 and 6 nm in size can be viewed as a three-dimensional packing of such clusters. Adjacent clusters are connected by a typical pair of edges, corners or faces, or by a combination-shared octahedra, forming oxo bridges in the bare cluster (Figure 2C) (Michel et al., 2007). The Fe-Fe distance depends on the architecture, with the corner-sharing arrangement having the longest (3.39–3.70 Å) and the face-sharing arrangement having the shortest distance (2.88 Å; Figure 2D) (Manceau and Combes, 1988; Cornell and Schwertmann, 2003).

The metal architecture of iron oxide colloids is susceptible to changes in the environment, including exposure to oxygen, reactive oxygen species, light, nitrate, ferrous or ferric irons, and phosphorus (Usman et al., 2018; Kappler et al., 2021). For instance, solar irradiation promotes a photo-oxidation process, even in the absence of oxygen (Braterman et al., 1983), triggering the transformation of Fht into Goe (Shu et al., 2019). Superoxide radicals were suggested to act as primary oxidants for Fe^2+^ under acidic conditions promoting the formation of iron oxide colloids (Shu et al., 2022). It has also been demonstrated that ferric oxyhydroxides such as Fht, Lep or Goe can be transformed into Mag when reacted with ferrous iron under alkaline conditions over time (Usman et al., 2012). Mag colloids are capable of converting into Mah, not only via oxidation by oxygen, various ions and/or ETs through the solid–solution interface (Jolivet and Tronc, 1988), but also through interaction with bacteria (Auffan et al., 2008). A similar transformation of the iron architecture has also been observed when Hem interacts with the iron-reducing bacterial strain *Shewanella oneidensis* MR-1 (Luo et al., 2017). Raman spectroscopy and analysis of magnetic properties reveal that this bacterial strain can transform the crystalline structure of Hem colloids from a hexagonal to a cubic system through microbial, extracellular ET. This transformation can also be monitored using electron paramagnetic resonance (EPR) spectroscopy, which shows that changes in the crystalline structure of Fe^2+^ lead to the biotransformation of Hem into Mag (Luo et al., 2017).

**FIGURE 3:**
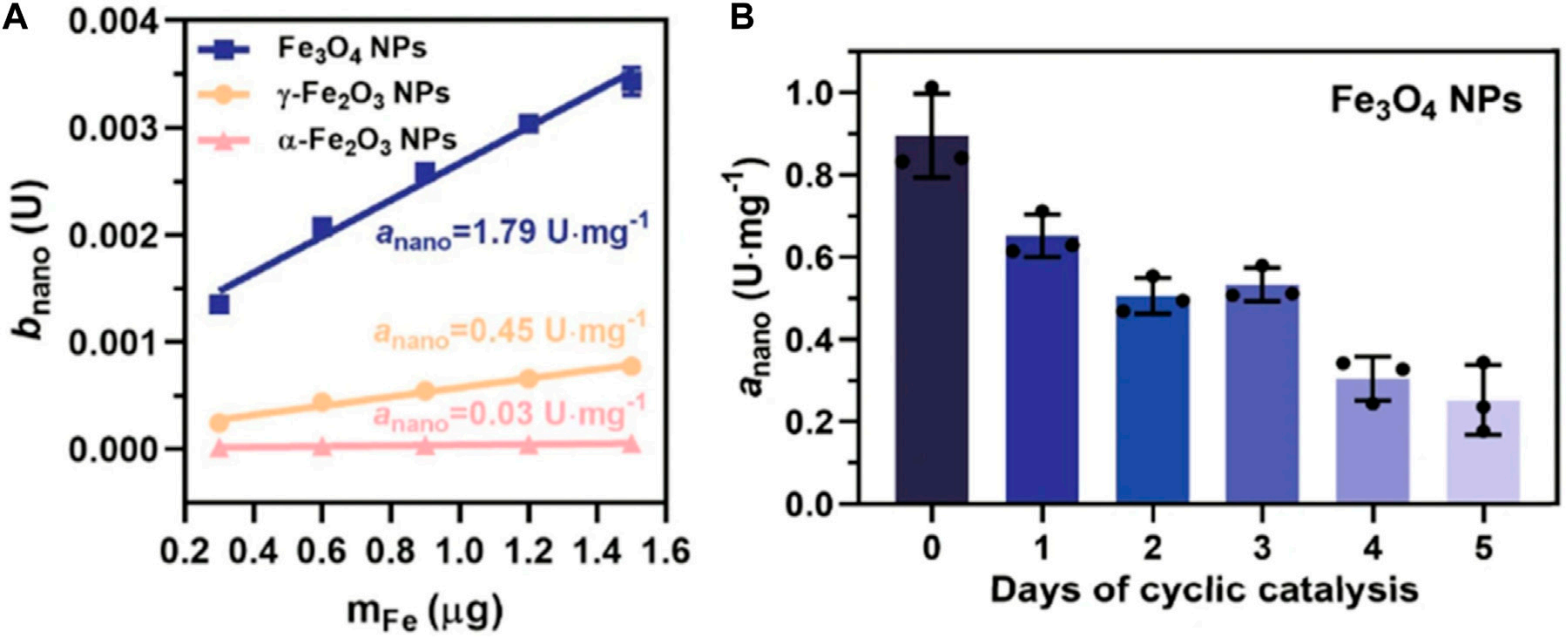
Schematic diagram of the catalytic mechanism of the activity of inorganic POD (Dong et al., 2022) **(A)** The specific POD-like activity (*a*_nano_) of Mag (Fe_3_O_4_), Mah (γ-Fe_2_O_3_) and Hem (α-Fe_2_O_3_), measured with TMB as colorimetric substrate. **(B)** Kinetic study of *a*_nano_ values of Fe_3_O_4_ NPs with the days of cyclic catalytic reaction. U is defined as 1 μmol/min for enzyme activity. Error bars represent standard deviation from three independent measurements. Reprinted from Dong et al., 2022.

The changes in the internal atomic structure of nanocolloids play an important role in their reactivity. For example, near-spherical Mag NPs with an average diameter of 10.16 ± 0.12 nm, gradually lose POD-like activity during their transformation from Mag to Mah. This transformation interferes with the rate of the ET at the surface of these nanocolloids (Figure 3) (Dong et al., 2022). The specific POD-like activity (*a*_nano_) of Mag, Mah and Hem NPs are 1.79, 0.45 and 0.03 Umg^−1^, respectively (Figure 3A) (Dong et al., 2022). However, the values of *a*_nano_ of Mag significantly decrease over time (Figure 3B) (Dong et al., 2022). Changes in the metal architecture are not limited to the colloid surface as the interior Fe^2+^ of Mag NPs are also gradually oxidized during prolonged reaction times. As a result, the catalytic activity of recovered NPs also gradually decreases concomitantly with an increase in their oxidation state (Dong et al., 2022). It has been proposed that ET to the surface via Fe^2+^-O-Fe^3+^ chains may enable the regeneration of surface Fe^2+^, thereby sustaining POD-like catalytic activity. The efficiency of this step has been proposed as the rate-limiting factor in NP-catalyzed reactions (Dong et al., 2022). Keep in mind that inorganic NP structures are not rigid and unchanging entities. Instead, they dynamically respond to a myriad of external influences, including both abiotic and biotic factors, as well as catalytic processing. These factors significantly impact the behavior of biocatalysts and have implications for the importance of metal center stabilization in the evolution of proteins.

## 3 Electron transfer mechanisms in inorganic iron oxide and iron sulfide nanocolloids

In the previous section we focused on the connection between architecture and catalytic activity and how NPs can change their architecture and hence also their activity. Here, we concentrate on the electronic properties of catalytically active colloids, their dependence on structure and their implications for catalysis or chemical transformations.

In inorganic colloids, the band gap (*i.e.,* the energy required to remove an electron from its valence shell) plays a significant role in ET processes and hence catalytic activity. The band gap is inherently related to the electron configuration, structural characteristics and charge ordering (*i.e.,* the long-range order of different metal oxidation states within the crystal lattice of the colloids (Verwey, 1939)). A narrow band gap facilitates electron hopping, a phenomenon where electrons spontaneously move between localized states or sites within a material through a series of intermediate states. This efficient movement of electrons contributes to the material’s catalytic activity by promoting effective ET processes.

On the other hand, proteins, DNA and RNA also exhibit electron hopping due to their own unique structural and chemical properties (Giese, 2018). The study of the connection between ET and conductivity at the molecular level, particularly the interplay between solid-state physics and bioinorganic chemistry, is an area of active research (Bostick et al., 2018; Mostajabi Sarhangi and Matyushov, 2023). The occurrence of electron hopping has been suggested for various iron oxide colloids, such as Mag (Skomurski et al., 2010), Fht (Alexandrov and Rosso, 2014), Goe (Zarzycki et al., 2015), green rust (Wander et al., 2007) and Hem (Iordanova et al., 2005; Kerisit and Rosso, 2006). Experimental observations have confirmed electron hopping on the surfaces of Fht (Katz et al., 2012), Hem (Carneiro et al., 2017; Husek et al., 2017), and Mah (Ibrahim et al., 2018).

**FIGURE 4:**
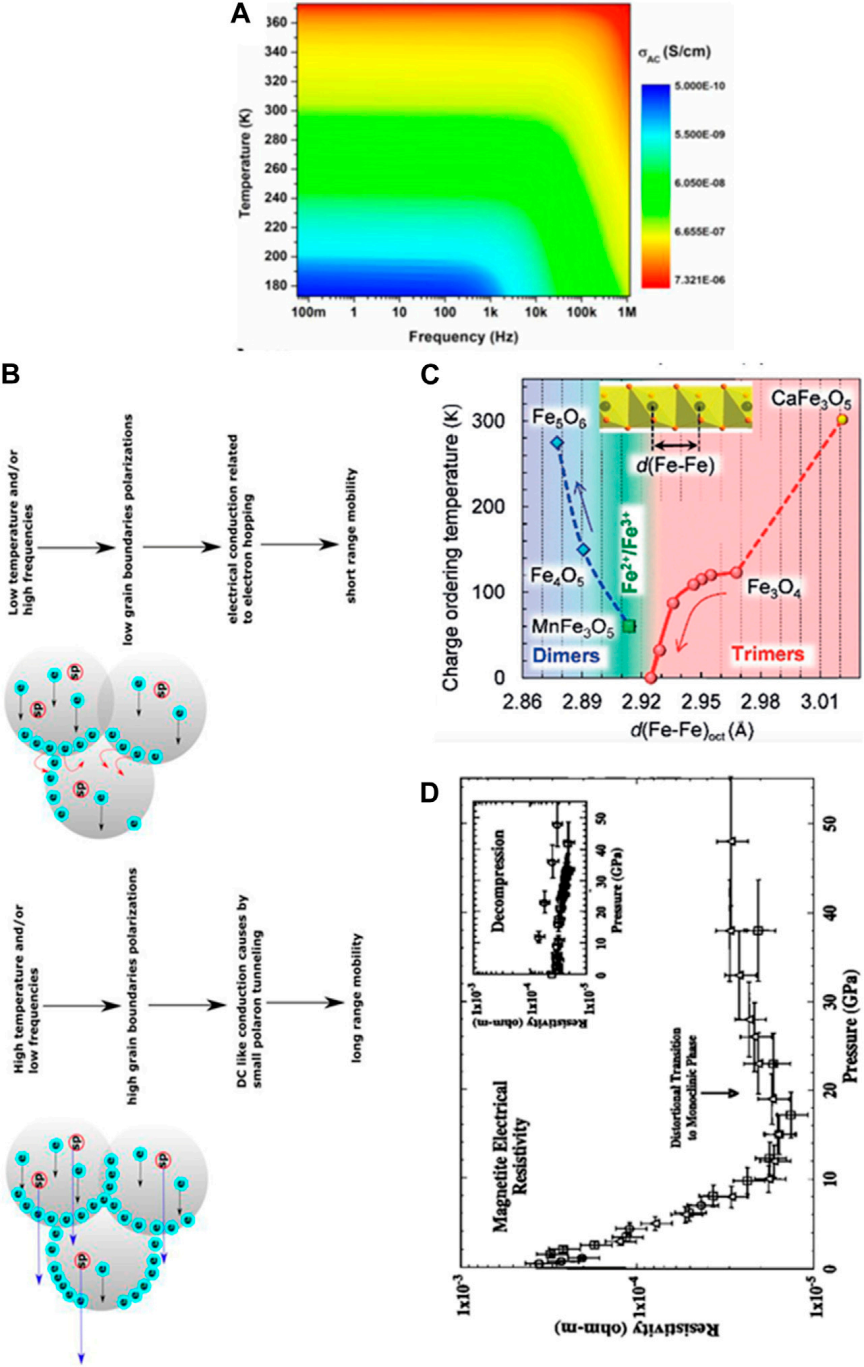
Structure and electrical characteristics of inorganic iron oxide colloids. **(A)** Surface plot of AC conductivity of Mag as a function of temperature and frequency. **(B)** Two conduction mechanisms in different temperature and frequency regions (Reprinted from Radoń et al., 2018). **(C)** Dependence of the charge-ordering transition temperature for Fe_5_O_6_, Fe_4_O_5_, MnFe_3_O_5_, Fe_3_O_4_, and CaFe_3_O_5_ on the minimal Fe−Fe distances in their octahedral iron chains (Reprinted from Ovsyannikov et al., 2020). **(D)** Response of electronic resistivity of Mag under different pressures (Reprinted with permission from Morris and Williams, 1997, Copyright ^©^ 1997, John Wiley and Sons).

The electrical conductivity of Mag nanocolloids, for instance, is affected by alternating current (AC) frequency and temperature, as shown in Figure 4A (Radoń et al., 2018). Conductivity dispersion as a function of AC frequencies is closely related to both long-range (conduction mechanism associated with grain boundaries) and short-range mobility (conduction mechanism associated within grains; Figure 4B). The blue arrow represents the tunnelling of small polarons, the solid red arrow represents electron hopping, and the black arrow represents electrons moving between Fe^2+^ and Fe^3+^ ions in the crystal structure. At high temperatures and low frequencies, tunnelling of small polarons occurs, which is associated with the polarization of grain boundaries and manifests itself as long-range mobility (Figure 4B) (Radoń et al., 2018).

Alterations in the Fe-Fe distance in the octahedral chains of various iron oxide colloids can also affect the ability of electrons to hop or tunnel between ions, leading to changes in charge ordering that relates to electrical conductivity (Todo et al., 2001; Senn et al., 2012; Ovsyannikov et al., 2016; Hong et al., 2018; Ovsyannikov et al., 2018; Cassidy et al., 2019; Ovsyannikov et al., 2020) (Figure 4C). Similar effects on electronic properties under pressure (causing structural changes) have been reported for Mah, Hem and Foh NPs (Morris and Williams, 1997; Pasternak et al., 1999; Ohta et al., 2010; Ohta et al., 2012) (Figure 4D).

The electrical conductivity of iron oxide nanocolloids is also influenced by their concentration; specifically, in a Mag nanofluid with varying volume fractions, the electrical conductivity increases with increasing temperature and weight fraction (Jamilpanah et al., 2017). At 25°C, the electrical conductivity of the base fluid increased from 0.39 μS cm^-1^ to 2,419 μS cm^-1^ for a loading of 4 vol% iron oxide, which corresponds to an anomalous enhancement of over 6,000 fold.

**FIGURE 5:**
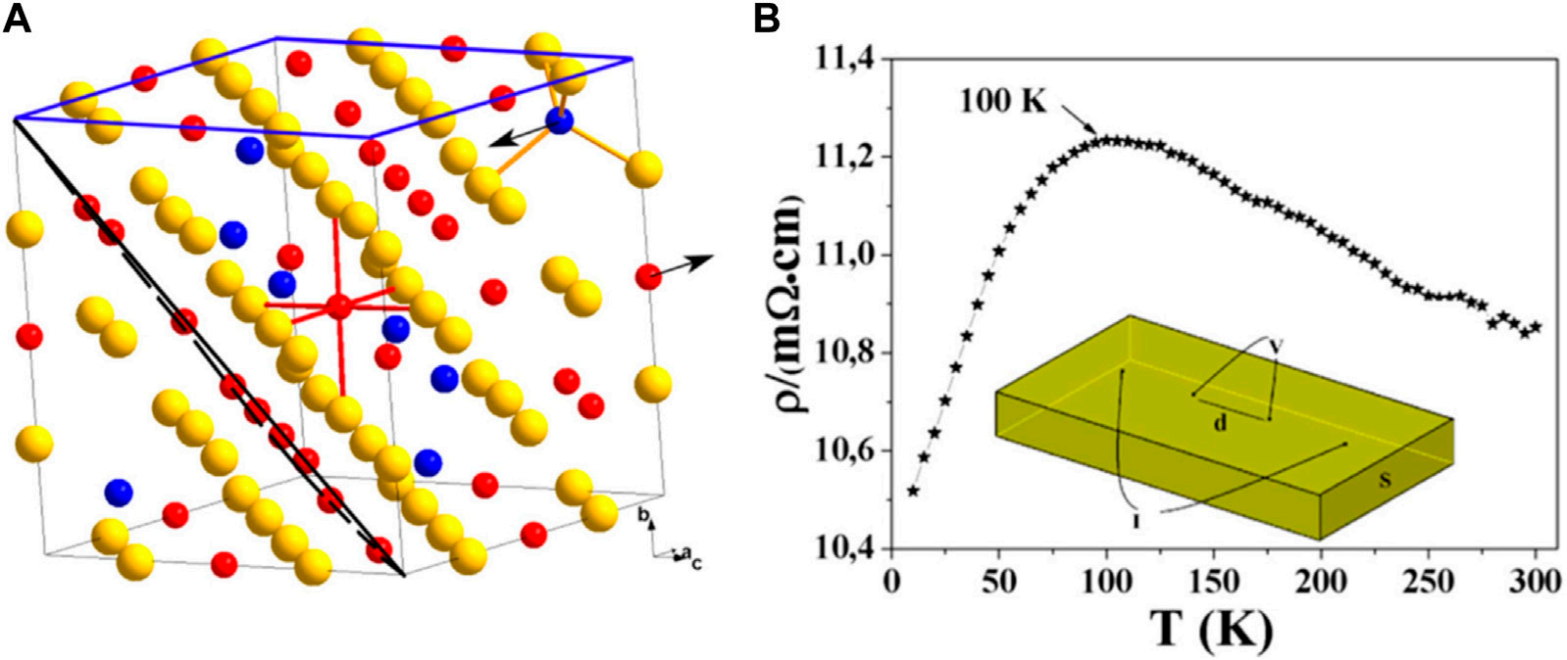
Structure and electronic properties of an Fe_3_S_4_ colloid. **(A)** Crystal structure of Fe_3_S_4_ with the (001) and (111) planes outlined in blue and black, respectively. Sulfur atoms (yellow spheres) form a cubic close-packed lattice: 1/8 of the tetrahedral A sites are occupied by Fe^3+^ (blue spheres) and 1/2 of the octahedral B sites are equally occupied by Fe^2+^ and Fe^3+^ (red spheres). The magnetic moments on the A and B sites are antiparallel and aligned along the [100] crystallographic axis (indicated by arrows). **(B)** Resistivity of Fe_3_S_4_ between 5 K and 300 K, and the corresponding contact geometry (inset). Reprinted with permission from Li et al., 2014, Copyright ^©^ 2014, ACS.

The ferrimagnetic iron sulfide greigite (Fe_3_S_4_) has an inverse spinel structure, consisting of both Fe^2+^ and Fe^3+^ centers in a 1:2 ratio. The spin magnetic moments of the Fe cations in the tetrahedral sites are oriented in the opposite direction to those in the octahedral sites (anti-ferromagnetic coupling), resulting in a net magnetization (Devey et al., 2009; Pattrick et al., 2017). Both metal sites have high-spin quantum numbers, and the mineral is a half-metal with an S vacancy structure and a magnetic moment of <4.0 μB per formula unit (Li et al., 2014) (Figure 5A). Fe^2+^-Fe^3+^ electron hopping occurs at the octahedral sites. When comparing the properties of Fe_3_S_4_ and Fe_3_O_4_, the mean charges for octahedral Fe are 1.0 e^−^ and 1.7 e^−^, respectively, while for tetrahedral Fe, they are 1.1 e^−^ and 1.8 e^−^, respectively. The value of magnetization of saturation (Ms) in sulfides is slightly less than that of oxides (Roldan et al., 2013) and the resistivity of sulfides is also less than that of oxides (Figure 5B) (Li et al., 2014).

Another iron sulfide example is pyrrhotite (Fe_1-*x*_S; with *x* varying between 0 and 0.13), which has a hexagonal crystal structure, where the metal ions are in an octahedral coordination environment and the anions in a trigonal prismatic arrangement. A crucial feature of this structure is the ability to omit metal atoms up to one in every eight (1/8), thereby creating iron vacancies. One such structure is pyrrhotite-4C (Fe_7_S_8_) (Sakkopoulos et al., 1984; Sagnotti, 2007). The Fe deficiency affects both the crystallographic and magnetic structures. The ordering of the Fe vacancies leads to an alternating arrangement of partially vacant and fully filled Fe layers, the hexagonal structure distorts to monoclinic and the magnetic ordering turns from antiferromagnetic to ferrimagnetic (Takele and Hearne, 1999; Roberts et al., 2018; Živković et al., 2021). Like in iron oxides, the structures of iron sulfide colloids also change with pressure (Takele and Hearne, 1999) or temperature (Roberts et al., 2018). The highly symmetrical structure of FeS results in an overall net zero magnetic moment across the unit cell. In contrast, the low symmetry structure of Fe_7_S_8_ exhibits ferrimagnetism due to the uncompensated magnetic moment in the iron-vacancy-rich layers. The vacancy-free sample (*x* = 0, troilite) has a metallic state in resistance and exhibiting superconductivity below 4.5 K (Lai et al., 2015). In contrast, for the samples with Fe vacancies (*x* ≥ 0.05), no superconductivity is observed, and the samples exhibit semiconducting behavior (Guo et al., 2017; Kuhn et al., 2017). Delocalized electrons in ultrathin Fe_7_S_8_ nanosheets facilitate ET as the d orbitals of Fe^2+^ and Fe^3+^ overlap. This electronic property is critical for its utilization as a catalyst, making ultrathin pyrrhotite nanosheets a very efficient Fe-based electrocatalysts for water oxidation (Chen et al., 2017).

In summary, the crystal structures of iron oxides and sulfides significantly influences their electrical properties, which are determined by the coordination of iron with oxygen or sulfur and the corresponding electronic configurations. The electron configuration and coordination of iron with oxygen or sulfur are crucial factors in determining the metal architecture of colloids, which contributes to their unique properties, including size and shape (Wu et al., 1997; Cornell and Schwertmann, 2003; Grau-Crespo et al., 2010; Yu et al., 2012; Erlebach et al., 2015; Noh et al., 2015; Huang X. et al., 2016; Li et al., 2017; Jian et al., 2019; Paidi et al., 2021). Iron oxides and sulfides exhibit semiconductor behavior with low band gaps, facilitating ET. The non-uniform coordination of Fe 3d electrons with oxygen or sulfur atoms yields a material that can induce intrinsic spontaneous electron hopping at non-uniform octahedral surface sites.

## 4 The relevance of inorganic oxidoreductase activity in biological systems

Iron oxide and sulfide nanocolloids are abundant on Earth and can be found in diverse habitats, including soils, water, rocks and living organisms (Cornell and Schwertmann, 2003; Jolivet et al., 2006; Rickard and Luther, 2007; Sagnotti, 2007; Navrotsky et al., 2008; Konishi et al., 2012; Guo and Barnard, 2013; Posth et al., 2014; Maher, 2016; Claudio et al., 2017; Yuan et al., 2020; Huang, 2022b). These encompass diverse environments such as high pH hydrothermal vents (Lough et al., 2019; Yücel et al., 2021), ice sheets (Hawkings et al., 2014), fly ash and street dust (Yang et al., 2016b; Gonet and Maher, 2019). Remarkably, they are also found in magnetosomes from Magnetotactic Bacteria (MTB) (Pósfai et al., 2013; Uebe and Schüler, 2016; Goswami et al., 2022), as well as in other biogenic iron minerals (Posth et al., 2014). These nanocolloids form through various mechanisms (Guo and Barnard, 2013), resulting in a range of sizes, shapes, and structures (Xie et al., 2018). Iron sulfide nanocolloids are prevalent in hydrothermal vent plumes (Findlay et al., 2019; Yücel et al., 2021) and can be found in many marine sediments (Rickard and Luther, 2007; Gu X. et al., 2020; Subramani et al., 2020). Geological evidence indicates that secondary pyrrhotite, pyrite, greigite, mackinawite and green rust (fougerite) may have existed as nanocolloids during the Hadean and early Archean era, a time period that predates and overlaps with the emergence of proteins and primitive life forms (Holland, 2007; Raiswell and Canfield, 2012; Bekker et al., 2013; Catling, 2013; Halevy et al., 2017; Goswami et al., 2022). Notably, simulations conducted in origin-of-life reactors produced pyrrhotite, pyrite and mackinawite (Herschy et al., 2014; White et al., 2015; White et al., 2020). Fe_2_O_3_ NPs obtained from PVC dichlorination residues and iron chips treated with subcritical water exhibit inherent peroxidase-like properties (Qi et al., 2023). It is anticipated that any iron oxide NPs with the same metal architecture continue to function as biocatalysts, a realization yet to be fully acknowledged.

During the Archean era, the primitive atmosphere was mainly composed of nitrogen, carbon monoxide, carbon dioxide and methane (Kasting et al., 1984; Lyons et al., 2014) but also potentially low levels of O_2_ and H_2_O_2_ (Zuo and Deng, 1999; Borda et al., 2001; Lee et al., 2019; He et al., 2021; Jenkins et al., 2021; Stone et al., 2022). The oceans contained Fe^2+^ and transition metal oxide, sulfide and potentially selenide nanocolloids (Braterman et al., 1983; Holland, 2007; Nitschke and Russell, 2009; Bekker et al., 2013; Shu et al., 2019; Shu et al., 2022). In order to broadly address the roles of metal nano-to sub-micro-sized catalysts on life and the habitability of Earth, we need to consider the basic requirements for life, *i.e.,* all cells need a source of energy and are composed of water, organic carbon molecules and essential elements (hydrogen, oxygen, nitrogen, phosphorus, and sulfur). The occurrence of complex organic carbon molecules and essential elements in the materials that formed the proto solar system cloud suggests that these materials, and possibly mineral catalysts were ubiquitous. Endogenous sources of organic carbon included the primordial, slightly reducing atmosphere (Miller, 1953; Johnson et al., 2008) and active hydrothermal systems producing organic carbon *via* Fischer Tropsch synthesis, *e.g.,* the Rainbow ultramafic hydrothermal system on the Mid Atlantic Ridge (Russell et al., 2010). A significant number of organic molecules (and other volatiles, such as water) were also delivered from extraterrestrial sources, *e.g.,* carbonaceous chondrites, containing up to 5% organic carbon (Sephton and Botta, 2008; Potiszil et al., 2023). Habitable conditions are defined by the sum of the physical and chemical conditions that support the presence of liquid water at the surface of a planetary body. Under standard (Earth) temperature and pressure, the occurrence of liquid water and catalytic activity could have occurred over a broad range of temperatures (−15°C–100°C) and salinity (freshwater to saturated brines), conditions that are considered to be extreme on Earth today. An origin of life under these extreme conditions is thought to be aligned with the Archaeal domain (Woese et al., 1990), which is dominated by prokaryotes that thrive in anaerobic (methanogen), thermophilic (high temperature) and halophilic (salt loving) extreme environmental conditions, common on early Earth. Anaerobic (reducing) mineral catalysts, *e.g.,* iron sulfides, would have affected the geochemistry of this early Earth, producing substrates for early life from the late heavy bombardment ∼3.9 billion years ago (Gomes et al., 2005) and continuing through the origin of life era, about 3.5 billion years ago (Westall and Southam, 2006), until the Great Oxidation Event (GOE) beginning from ∼ 2.5 billion years ago. During this time, the Earth possessed an anaerobic, habitable environment with < 0.2% of the present atmospheric oxygenic levels (Catling and Claire, 2005) and that was significantly hotter (Knauth and Lowe, 2003; Cavalazzi et al., 2021) and more volcanically/hydrothermally active (Hofmann and Bolhar, 2007) than most contemporary systems. The low levels of reactive oxygen produced by photolysis (Kasting, 1993) relative to the abundance of reduced chemical species would have resulted in a correspondingly reducing chemistry for the hydrosphere and lithosphere, though some transient metal oxides, *i.e.,* metal oxide colloids, may have been formed and been ‘active’ in this system. From the GOE forward, Earth has had variable, but more oxidizing conditions, increasing the diversity of catalytic nanomaterials, *e.g.,* partially oxidizing (such as Mag) to fully oxidizing materials (such as 2L-Fht or 6L-Fht), as well as the ‘earlier’ reducing mineral catalysts.

Ever since the GOE, the presence of hydrogen peroxide and free radicals in the environment has been a challenge for living cells, in particularly anaerobic bacteria, which do not have efficient enzymatic detoxification strategies (Dröge, 2002; Halliwell, 2006; Ślesak et al., 2007; Sies, 2017; Taverne et al., 2018; Taverne et al., 2020). ROS, such as hydrogen peroxide, are byproducts of normal metabolic processes in cells and can cause oxidative damage to cellular components such as DNA, proteins and lipids. The Snowball Earth and GOE periods may have contributed to an increase in atmospheric hydrogen peroxide levels, potentially leading to detrimental effects such as mutations, cell death and other adverse impacts on organism survival and evolution (Liang et al., 2006). It has been speculated that essential enzymes like SOD, CAT and POD may have existed prior to the GOE (Castresana et al., 1994; Zelko et al., 2002; Slesak et al., 2012; Zámocký et al., 2012; Inupakutika et al., 2016; Ślesak et al., 2016; Case, 2017; Olson et al., 2017). Furthermore, their activities may have been complemented/augmented by iron oxide and sulfide nanocolloids, thus mitigating the detrimental effects of ROSs (Huang, 2018; Huang, 2022a). Such activities are found in all domains of life, including obligate anaerobes, suggesting that the need for such protection prevailed even in anaerobic environments (Runnegar, 1991; Castresana and Saraste, 1995; Lenton, 2003; Neubeck and Freund, 2020). These suggestions are consistent with the hypothesis that inorganic iron oxide or sulfide colloids with intrinsic oxidoreductase activity and/or which promote spontaneous electron hopping may have been crucial to establish and enhance biological reaction rates at the onset of biological evolution. Remarkably, iron oxide nanocolloids, such as Mag and ferrihydrite, can directly cross lipid bilayers and enter the cytoplasm and other cellular compartments of eukaryotic cells without damaging the plasma membrane (Zanella et al., 2017; Chilom et al., 2020).

As described above, some microorganisms are able to trigger architectural changes of iron colloids, especially the nanocolloids and consequently can also alter their catalytic activity (*e.g., E. coli* or *S. oneidensis* MR-1 (Luo et al., 2017)). Another example is *Trichoderma guizhouense*; incubation of Mag nanocolloids with this fungus leads to a significant increase in their POD-like activity (∼2.4-fold increase) (Chi et al., 2021). These observations demonstrate that nature is not only able to utilize inorganic colloids but to also optimize their oxidoreductase activity through modifications of their metal architecture. Further, recent research has demonstrated that the ET rate of inorganic iron oxide NPs can also be augmented by small molecules such as amino acids or nucleotides (Fan et al., 2017; Niu et al., 2018; Wu W. et al., 2019; Chen J. et al., 2020; Han et al., 2020; Niu et al., 2020; Vallabani et al., 2020; Xu W. et al., 2021; Geng et al., 2021; Han et al., 2021; Sun et al., 2021; Wang et al., 2023). It is widely accepted that primitive precursors of these molecules emerged early during Earth’s prebiotic evolution (Miller, 1953; OrÓ, 1961; Ferus et al., 2017; Frenkel-Pinter et al., 2022), contributing to the development of life, including the formation of proteins, DNA and RNA. For instance, the complexation of Mag NPs with the amino acid histidine (His) improves their *K*_m_ for H_2_O_2_ over ten-fold (from 459 mM to 38 mM) and increases their catalytic efficiency (*k*_cat_/*K*_m_) up to 20-fold (from 0.68×10^6^ s^-1^M^-1^ to 14.2×10^6^ s^-1^M^-1^) (Fan et al., 2017). For comparison, the corresponding values for the enzyme HRP are 10.4 mM and 0.29×10^6^ s^-1^M^-1^ (Fan et al., 2017). The addition of organic functional groups, such as amino acids or nucleotides, to inorganic oxidoreductases likely played a vital role in stabilizing the structure of the early catalysts during evolution (Huang, 2022a), while also promoting electron tunneling (*via* super-exchange) and hopping (Halpern and Orgel, 1960; Hopfield, 1974; Marcus and Sutin, 1985; Warren et al., 2012; Gray and Winkler, 2015; 2021). Notably, electrons can tunnel through peptides in microseconds over distances of 15–20 Å, a phenomenon assisted by aromatic side chains of amino acids such as tryptophan (Trp) and tyrosine (Tyr) (Gray and Winkler, 2021).

In the study of ET in proteins, attention is given to factors such as the amino acid composition, overall fold and hydrogen bonds (Dixon and Lipscomb, 1976; Dwyer, 2006; Warren et al., 2012; Berstis et al., 2015; Gray and Winkler, 2015; Sepunaru et al., 2015; Gray and Winkler, 2021). Similarly, evolutionary studies of metalloenzymes have mostly focused on their protein folds (Grishin, 2001; Raanan et al., 2020), and less so on their metal centers (Holm et al., 1996; Drennan and Peters, 2003). Recently, it was proposed that metalloenzymes, including ribozymes (Pyle, 1993), may be considered as functionalized nanomaterials, in which the metal architecture serves as an active center that has been stabilized over time by amino acids and nucleic acids (Huang, 2022a). This line of thought is supported by the fact that certain inorganic colloids exhibit enzyme-like properties and with similar metal architectures as the active sites of enzymes such as POD, OXD, CAT or SOD, but also purple acid phosphatase (Mitić et al., 2006; Huang and Zhang, 2007; 2012; Schenk et al., 2013; Huang, 2018; 2019), haloperoxidase (André et al., 2011; Natalio et al., 2012; Leblanc et al., 2015; Chen, 2022) and sulfite-oxidizing enzymes (Hille et al., 2014; Ragg et al., 2014; Kappler and Enemark, 2015). It is important to note that metalloenzymes have highly complex and fine-tuned structures that have evolved over time, incorporating both a metal center and specific amino acid side chains that contribute to their fold, tertiary/quaternary structures, as well as their ability to confer catalytic activity.

Another poignant example that illustrates the evolution of a metalloenzyme starting from an inorganic core is ferredoxin, an Fe-S-containing protein that was identified as an essential component of photosynthesis well before its amino acid sequence was known (Eck and Dayhoff, 1966). Indeed, Fe-S clusters were present in the last universal common ancestor (LUCA) of life on Earth, where they may have been used for various purposes, including ET and redox reactions (Weiss et al., 2016). This hypothesis is supported by research on hydrothermal vents that mimic conditions that may have been present at the onset of living organisms (Baross and Hoffman, 1985; Russell and Hall, 1997; Nitschke and Russell, 2009). The Fe-S clusters in proteins exhibit considerable similarity to various iron sulfides (Zhao et al., 2020; McGuinness et al., 2022). Relevant examples include eukaryotic ferredoxins and Rieske proteins that contain a Fe-S cluster with two Fe and two S atoms forming a 2Fe-2S diamond (Holden et al., 1994; Gurbiel et al., 1996), while higher potential iron-sulfur proteins and iron regulatory proteins (IRPs) use four Fe and four S atoms to form a cubic 4Fe-4S cluster (Breiter et al., 1991; Solomon et al., 2000; Dupuy et al., 2006; Imlay, 2006). Rubredoxin, on the other hand, possesses a single iron atom coordinated by four equidistant sulfur atoms, forming a 1Fe-4S tetrahedron (Adman et al., 1991; Liu et al., 2015). Furthermore, although rare, 3Fe-3S (Bruschi and Guerlesquin, 1988) and 6Fe-6S clusters (Stokkermans et al., 1992) are also observed, demonstrating the architectural diversity of iron sulfide minerals. How these different clusters evolved in protein environments remains obscure. However, it is worth noting that iron sulfides with a single iron atom coordinated by four equidistant sulfur atoms exhibit superconductivity (Lai et al., 2015; Guo et al., 2017; Kuhn et al., 2017) and high inorganic oxidoreductase activity (Dai et al., 2009; Dutta et al., 2012b; Yao et al., 2013; Ding et al., 2016; Xu et al., 2018), suggesting that they may have played important roles in the biochemistry of LUCA and thus the evolution of FeS-containing proteins.

The catalytic activity of cubane-type Fe_4_S_4_ clusters in metalloproteins like biotin synthase (Reyda et al., 2009), aconitase (Castro et al., 2019), and (E)-4-hydroxy-3-methylbut-2-enyl pyrophosphate reductase (IspH) (Span et al., 2012), as well as in synthetic M_4_S_4_ clusters for various reactions, illustrates their possible role in the emergence of life and the formation of organic compounds from inorganic precursors (Seino and Hidai, 2011). Recent studies show that Fe–S clusters with low-valent Fe^1+^ centers can adopt a wide range of electronic configurations, crucial for their catalytic activity (Brown et al., 2022). CO binding to a synthetic [Fe_4_S_4_]^0^ cluster with N-heterocyclic carbene ligands triggers the generation of Fe^1+^ centers through intracluster ET, demonstrating the Fe-S clusters’ ability to facilitate ET in redox reactions. CO binding to an [Fe_4_S_4_]^+^ cluster induces electron delocalization with a neighboring Fe site, resulting in a mixed-valent Fe^1.5+^Fe^2.5+^ pair, thus enabling the activation of C–O bonds without highly negative redox states (Brown et al., 2022). Metalloproteins with Fe_4_S_4_ clusters catalyze CO and CO_2_ reduction to hydrocarbons (alkanes/alkenes) (Lee et al., 2010; Rebelein et al., 2015; Waser et al., 2023), significant in context of early Earth’s life origins.

Pyruvate is a central metabolite in Archaea, Bacteria and Eukarya kingdoms, where iron-sulfur enzymes connect pyruvate to carbon fixation pathways and thioester biochemistry (De Duve, 1991; Berg et al., 2010). The FeS/S/FeS_2_ system catalyzes hydroxyl acids and keto acids interconversion (Wang et al., 2011). Recent studies show natural iron sulfide pyrrhotite acting as an oxidoreductase catalyst in pyruvic acid to lactic acid conversion (De Aldecoa et al., 2013) and CO_2_ reduction (Mitchell et al., 2021). Although these studies lack detailed kinetic data for the NPs’ inorganic oxidoreductase activity, they align with Wächtershäuser’s mineral surface study focusing on the iron-sulfur world and its relevance to evolutionary biochemistry (Wächtershäuser, 1988, 1990, 1992).

Contemporary biological systems demonstrate the versatile applications of inorganic NPs across various fields. In biomedicine, iron oxide NPs have shown promise for therapeutic and diagnostic purposes. For example, ferrihydrite NPs exhibiting CAT-like activity, were found to enhance the effectiveness of radiotherapy (Zhang R. et al., 2021), while magnetoferritin NPs have been employed for targeting and visualizing tumor tissues (Fan et al., 2012). Additionally, dietary iron oxide NPs with CAT activity has been shown to mitigate neurodegeneration in a Drosophila-Alzheimer’s disease model (Zhang et al., 2016). These findings highlight the potential of iron oxide NPs in addressing aging-related metabolic disorders and neurodegenerative diseases associated with increased ROS production. In agriculture, inorganic NPs have been studied for their effects on plant growth and nutrient uptake. Recent research has indicated their role in enhancing nitrogen fixation, yield, and nutritional quality of soybeans (Cao et al., 2022). Furthermore, foliar application of iron oxide NPs has been observed to stimulate plant growth and act as a defense response against plant viruses (Cai et al., 2020). These findings underscore the potential of inorganic NPs in sustainable agriculture practices. Moreover, inorganic NPs have shown promise in environmental applications, particularly in remediation and pollution control. For instance, green-synthesized magnetite NPs have demonstrated antifungal potential in protecting plants against wilt infection (Ashraf et al., 2022). They have also been effective in mitigating the harmful effects of heavy metal contamination in plants, such as reducing cadmium accumulation in rice biomass (Rizwan et al., 2019; Sarraf et al., 2022; Lu et al., 2023). These applications highlight the diverse potential of inorganic NPs in addressing environmental challenges and contributing to sustainable environmental management.

In summary, the multifaceted applications of inorganic NPs span biomedicine, agriculture and environmental remediation. Leveraging the functional properties of NPs facilitate a growing number of innovative solutions for a wide range of challenges, from improving human health to enhancing agricultural productivity and addressing environmental pollution.

## 5 Conclusion

Inorganic ‘biocatalysts’ were crucial components of prebiotic chemical reactions related to the emergence of life (Bernal, 1951; Williams, 1981; Cairns-Smith, 1985; Williams, 2003), and remain central to many contemporary biological processes. The “metabolism-first” model for the emergence of life posits the development of metabolic networks prior to the emergence of genetic material (Oparin, 1938; Bernal, 1951). This model considers key inorganic processes, such as pyrite formation and serpentinization (Russell and Hall, 1997; Russell et al., 2010; Russell, 2023), which may have played a role in early biochemical reactions due to their surface properties and potential catalytic capabilities (Wächtershäuser, 1992).

Recent perspectives, supported by the discovery of nanocolloidal mineral biocatalyst activity, have shed light on the significance of metal architectures in catalysis, particularly in biological processes (Huang, 2022a). Laboratory studies have demonstrated that inorganic iron-oxide, -sulfide, and -selenide NPs exhibit unique oxidoreductase activity, arising from their metal architecture rather than solely their surface properties. ET and electron hopping within these NPs are influenced by the electronic structure of the metal ions and their coordination with oxygen, sulfur, or other elements, enhancing their oxidoreductase activity. The presence of these inorganic nanocolloids in early Earth environments suggests their involvement in crucial geological and chemical processes, including potential contributions to the first life and the evolution of biological systems.

The essential role of inorganic oxidoreductases in the emergence and evolution of life extends to their influence on the development and adaptation of living organisms over time. These catalysts have been fundamental in shaping the metabolic pathways that form the basis of cellular energy production and utilization using ET. By catalyzing key redox reactions, inorganic oxidoreductases have enabled organisms to efficiently harness and utilize energy from their environments. Furthermore, inorganic oxidoreductases have been involved in biogeochemical cycles that have shaped the availability and cycling of essential elements like carbon, oxygen, phosphorus, sulfur, iron, manganese, and chromium, as well as trace metals such as uranium, in the environment. These cycles play a crucial role in regulating the distribution and cycling of these elements between the atmosphere, lithosphere, hydrosphere, and biosphere.

The discovery of inorganic nano-sized catalysts substantiates the significance of metal architecture in biocatalysts from the onset of the evolution of life on our planet. Furthermore, enhancing our understanding of the contributions of inorganic nanocolloids to the evolution of life may also deepen our understanding of Earth’s ecosystems and their interconnectedness. These inorganic nanocolloids and their catalytic activity may have applications in various fields, including biomedicine, agriculture, and environmental science, owing to their stability and high catalytic efficiency.

## Figures and Tables

**Table:** 

•O2*	superoxide radicals
**α-Fe**_**2**_ **O**_**3**_	Hematite
**γ-Fe**_**2**_ **O**_**3**_	Maghemite
**2L-Fht**	2-line ferrihydrite
**6L-Fht**	6-line ferrihydrite
**AKA**	akageneite
**AOx**	Alcohol oxidase
**ATP**	adenosine triphosphate
**CAT**	Catalase
**CeO**_**2**_	Ceria
**CeVO**_**4**_	Cerium vanadate
**Cyt c**	Cytochrome c
**DAB**	diazoaminobenzene
**DET**	Direct electron transfer
**DFT**	Density function theory
**DNA**	Deoxyribonucleic acid
**DPD**	N, N-diethyl-1,4-phenylenediamine
**EPR**	Electron paramagnetic resonance
**ET**	Electron transfer
**ETp**	Electron transport
**Fe**_**1−x**_ **S**	Pyrrhotite
**FeO**	Wustiteite
**Fe-O**	Iron is bonded with oxygen atoms, such as iron oxides (e.g., hematite, magnetite) or other iron-oxygen complexes
**Fe**_**3**_ **O**_**4**_	Magnetite
**Fe**_**3**_ **S**_**4**_	Greigite
**Fe**_**9**_ **S**_**11**_	Smythite
**FeS**	Mackinawite
**Fe-S**	Iron is bonded with sulfur atoms, such as iron sulfides (e.g., pyrite, Pyrrhotite) or other iron-sulfur complexes
**FeS**_**2**_ **m**	Marcasite
**FeS**_**2**_ **p**	Pyrite
**Foh**	Feroxyhyte
**Goe**	Goethite
**GOE**	Great oxidation event
**Gox**	Glucose oxidase
**GPx**	Glutathione peroxidase
**H**_**2**_ **O**_**2**_	Hydrogen peroxide
**His**	Histidine
**HRP**	Horseradish peroxidase
**HRTEM**	High-resolution transmission electron microscopy
**IspH**	(E)-4-hydroxy-3-methylbut-2-enyl pyrophosphate reductase
**k**_**cat**_	Catalytic constant
**K**_**m**_	Michaelis-Menten constants
**Lc**	Laccase
**Lep**	lepidocrocite
**LUCA**	Last universal common ancestor
**MoS**_**2**_	Molybdenum disulfide
**Ms**	Magnetization of saturation
**MTB**	Magnetotactic bacteria
**NEXAFS**	Near Edge X-ray Absorption Fine Structure
**NPs**	Nanoparticles
**NWs**	Nanowires
**O**_**2**_	molecular oxygen
**OPD**	o-phenylenediamine
**OXD**	Oxidase
**POD**	Peroxidase
**ROS**	Reactive oxygen species
**Sch**	Schwertmannite
**SAED**	Selected area electron diffraction
**SEM**	Scanning electron microscope
**SOD**	Superoxide dismutase
**SOE**	Sulfite oxidase
**TEM**	Transmission electron microscopy
**TMB**	3,3,5,5-tetramethylbenzidine
**Trp**	tryptophan
**Tyr**	tyrosine
**V**_**2**_ **O**_**5**_	Vanadium pentoxide
**XAFS**	X-ray absorption fine spectroscopy

## Footnotes

1 The term “nanocolloids” is used here to highlight the importance of size (<100 nm) and high surface area-to-volume ratio in the enzyme-like activity of inorganic NPs. It is important to note that not all fine-grained mineral particles (colloids) exhibit the same enzyme-like activity. While the term “nanozyme” has become popular for describing NPs with enzyme-like activities, it is essential to understand its historical development. Initially, the term “nanozyme” referred to triazacyclonane/Zn^2+^-functionalized gold NPs as RNase mimics (Manea, F., Houillon, F.B., Pasquato, L., and Scrimin, P. 2004. Nanozymes: Gold-nanoparticle-based transphosphorylation catalysts. *Angew. Chem. Int. Ed*. 43**,** 6,165-6,169) Later, it was expanded to include nanomaterials with enzyme-like characteristics (Wei, H., and Wang, E. 2013. Nanomaterials with enzyme-like characteristics (nanozymes): Next-generation artificial enzymes. *Chem Soc Rev* 42**,** 6060-6093). Our review focuses on natural NPs, historically referred to as colloids, which encompass particles ranging from micro-to nanometer-sized dimensions. By using the term “nanocolloids,” we aim to underscore the natural origin and properties of NPs, distinguishing them from the artificial or engineered NPs often associated with nanozymes.
